# Vitamin C supplementation in patients with hypothyroidism requiring high-dose levothyroxine: a proof-of-concept pilot study

**DOI:** 10.3389/fendo.2025.1679835

**Published:** 2025-10-23

**Authors:** Adnan Agha, Bachar Afandi, Javed Yasin, Charu Sharma, Mohammad Hamdan Alshaer, Mouza Ali Saif Alshamsi, Dana Ebraheim Yaaqeib, Bayena Khamis Eshaq Alblooshi, Juma AlKaabi

**Affiliations:** ^1^ Department of Internal Medicine, College of Medicine and Health Sciences, United Arab Emirates University, Al Ain, United Arab Emirates; ^2^ Division of Endocrinology, Department of Internal Medicine, Tawam Hospital, Al Ain, United Arab Emirates; ^3^ Department of Genetics and Genomics, College of Medicine and Health Sciences, United Arab Emirates University, Al Ain, United Arab Emirates

**Keywords:** vitamin C, hypothyroidism, levothyroxine, proof-of-concept, pilot study, thyroid

## Abstract

**Background:**

Vitamin C supplementation may enhance the absorption of levothyroxine in patients with hypothyroidism. This proof-of-concept pilot study aimed to assess the frequency of vitamin C insufficiency and evaluate the feasibility and potential therapeutic signal of vitamin C supplementation in patients requiring high-dose levothyroxine.

**Methods:**

This two-phase study initially assessed vitamin C levels in 26 hypothyroid patients and 91 healthy controls. In phase two, a double-blind, randomized, placebo-controlled trial was conducted. Twelve patients were randomized, and 11 completed the study. Participants received either 1g daily vitamin C (n=6) or a near-matched pH placebo (n=5) for 16 weeks. Primary outcomes were changes in the Zulewski clinical score and thyroid-stimulating hormone (TSH) levels.

**Results:**

Vitamin C insufficiency was more frequent in hypothyroid patients (19.2%) versus controls (7.7%), though the difference in mean levels was not statistically significant (59.33 ± 24.62 µmol/L vs 73.12 ± 14.03 μmol/L in controls; p=0.21). In the RCT, the vitamin C group showed greater changes in Zulewski score (mean reduction 5.00 vs 1.40 points; difference 3.60, 95% CI: 1.88 to 5.32) and TSH levels (mean reduction 4.08 vs 2.35 mU/L; difference 1.73, 95% CI: -2.14 to 5.60) compared to placebo. However, the groups had significant baseline imbalances, notably in BMI (26.6 vs 43.4 kg/m²). After ANCOVA adjustment for baseline values, the between-group difference remained statistically significant for the Zulewski score (adjusted p=0.004) and marginally significant TSH (adjusted p=0.043). Primary biochemical outcome in this study was TSH rather than direct thyroid hormone measurement, as TSH represents the most sensitive biomarker for thyroid hormone adequacy in primary hypothyroidism and serves as the established therapeutic target in clinical guidelines.

**Conclusions:**

This proof-of-concept study demonstrates the feasibility of studying vitamin C supplementation in patients on high-dose levothyroxine and detects a therapeutic signal, particularly in clinical symptoms. However, the findings are limited by the very small sample size and severe baseline imbalances, precluding any conclusions on efficacy. These preliminary data justify the need for larger, well-controlled trials with stratified randomization to determine if this intervention translates into a clinically meaningful effect.

**Clinical Trial Registration:**

https://clinicaltrials.gov/study/NCT05733078, identifier NCT05733078.

## 1 Introduction

Thyroid disease can affect over a quarter of a billion individuals worldwide, with over 50% being unaware of their condition (1, 2). While autoimmune disorders are the commonest cause of thyroid dysfunction in iodine-sufficient regions, it is estimated that 2 billion individuals globally remain at risk for thyroid disease due to insufficient iodine intake (3). Autoimmune thyroid disorders are the most common cause of thyroid dysfunction in iodine-sufficient regions worldwide (4). Suboptimally treated or untreated hypothyroidism may lead to cognitive decline, dyslipidemia, hypertension, infertility, and cardiovascular and neuromuscular complications (5–7). The prevalence of hypothyroidism varies in the general population, with up to 5.3% of individuals experiencing overt hypothyroidism in Western countries (8), whereas approximately 10% of the global population may have subclinical hypothyroidism (9). In the Gulf region, although national studies providing the precise prevalence data on hypothyroidism are somewhat lacking, some cross-sectional screening studies suggest that its frequency may be as high as 5–10% (10, 11).

Levothyroxine is a synthetic form of thyroxine which is prescribed as a replacement monotherapy for hypothyroidism (12), and is primarily absorbed in the small intestine (13). Typically, the maximum daily levothyroxine requirement is 1.6 µg/kg body weight/day, which is sufficient to normalize thyroid-stimulating hormone (TSH) levels in most patients. However, approximately half the patients on replacement therapy may fail to achieve normal TSH levels and require further dose adjustments, possibly due to interference or malabsorption (13–15). In these patients, multiple dose adjustments and repeated diagnostic procedures not only increase healthcare costs but also elevate the risk of complications associated with sub-optimally controlled hypothyroidism (7, 16). As an alternative to increasing levothyroxine doses and achieving variable responses, adding vitamin C to levothyroxine therapy may enhance levothyroxine absorption and treatment efficacy, although this has previously been shown only in a specific subset of patients with gastritis (17). The effect of vitamin C on improving levothyroxine uptake has also been demonstrated over a short period in a non-randomized, non-controlled setting (18), but there have been no randomized controlled trials or any studies from Asian population to confirm these findings.

Ascorbate, discovered over a century ago as a potent reducing agent, was later identified as vitamin C and is an essential nutrient (19). Although most eukaryotes synthesize vitamin C endogenously from glucose, a few animals (such as bats, guinea pigs, birds and humans) lack the required enzyme, gulonolactone oxidase, rendering them completely dependent on dietary intake (20). Vitamin C has various biochemical roles, which includes being a major antioxidant and promoter of multiple essential enzymes like ketoglutarate-dependent dioxygenase and copper type-II monooxygenase, indicating potential role in stem cell proliferation (21). Humans have a limited capacity for storage of vitamin C, which makes it even more essential to have dietary intake and plasma vitamin C concentration of 40–75 µmol/L are considered adequate. Levels between 23–40 µmol/L indicate insufficiency, levels of 12–22 µmol/L suggest deficiency, and levels below 11 µmol/L are indicative of scurvy, which if left untreated is a fatal condition (22). Although a daily dietary intake of 200 mg of vitamin C may be sufficient for maintaining adequate levels, supplementation with more than 1000 mg per day is generally not recommended due to increased risk of kidney stone formation (23, 24). Regular consumption of more than 1 gram per day may elevate uric and oxalic acid levels in urine, that can potentially lead to kidney stone formation, especially in males (25). Studies in the western population on vitamin C levels indicate that nearly 1% of inhabitants exhibit vitamin C deficiency, while in lower socioeconomic status countries like India, vitamin C deficiency can be seen in nearly two-third of the sampled population, which has been attributed to low dietary intake. There have been no studies on published vitamin C levels in United Arab Emirates or in the neighboring Middle East countries in the literature, hence we wanted to explore this further.

The objective of the current study was twofold. First, we aimed to measure serum vitamin C levels in patients with hypothyroidism receiving the recommended daily dose of oral levothyroxine and compare them with those in a healthy young population without hypothyroidism to determine the presence or absence of vitamin C insufficiency in either group. Second, we sought to assess the biochemical and clinical responses in patients with hypothyroidism who were taking the recommended daily dose of oral levothyroxine (>1.6 µg/kg/day) upon supplementation with 1 g oral vitamin C daily compared with those taking a placebo.

## 2 Patients and methods

### 2.1 Study design and oversight

This proof-of-concept study was designed to establish feasibility and identify potential therapeutic signals of vitamin C supplementation in hypothyroidism. The two-phase design was conducted at Tawam Hospital and UAE University, Al Ain, United Arab Emirates, between January 2023 and December 2024. The study protocol was approved by the Tawam Human Research Ethics Committee (Approval #MF2058-2023-992; DOH Reference # DOH/CVDC/2023/492) and registered at ClinicalTrials.gov (NCT05733078). All participants provided written informed consent.

### 2.2 Phase 1: cross-sectional assessment of vitamin C status

#### 2.2.1 Participants

We recruited two groups for vitamin C assessment: Hypothyroid Group: Adult patients (≥18 years) with primary hypothyroidism on levothyroxine >1.6 µg/kg/day for ≥6 months from the Endocrinology Clinic. All patients had documented autoimmune hypothyroidism. Exclusion criteria included secondary hypothyroidism, thyroid surgery, known malabsorption disorders (e.g., celiac disease), gastric/intestinal surgery, patients on regular vitamin supplementation or acid suppression therapy, or TSH >20 mU/L. Control Group: Healthy university medical students without known medical conditions or regular medications. We acknowledge the significant age disparity with the patient group is a major limitation; this comparison was intended for internal hypothesis-generation only and its validity is severely limited.

#### 2.2.2 Procedures

Out of 36 eligible hypothyroid patients, 26 consented. A total of 91 controls were recruited. Blood samples were collected for serum vitamin C, T3, T4, and TSH levels.

### 2.3 Phase 2: pilot randomized controlled trial

#### 2.3.1 Trial design and participants

This was a 16-week, prospective, double-blind, placebo-controlled pilot RCT. Patients from Phase 1 were invited to participate. A total of 12 patients consented and were randomized (6 per group), a sample size consistent with established rules of thumb for pilot investigations (26).

#### 2.3.2 Randomization and blinding

Participants were randomized 1:1 using a computer-generated sequence (27, 28). To maintain blinding, the vitamin C and placebo preparations were dispensed in opaque, sealed containers by a research pharmacist. Investigators, participants, and outcome assessors remained blinded until the completion of the statistical analysis.

#### 2.3.3 Interventions

Participants took their assigned intervention daily with their levothyroxine dose. *Treatment Group:* 1g effervescent vitamin C daily for 16 weeks. *Placebo Group:* Effervescent oral rehydration salt (ORS) daily for 16 weeks. To minimize gastric pH as a confounding factor, we selected a vitamin C brand and an ORS brand with near-matched pH values (vitamin C: pH 4.86; ORS: pH 5.01; difference 0.15 pH).

#### 2.3.4 Rationale for not including positive control

This proof-of-concept pilot study did not include a traditional positive control group. In levothyroxine absorption research, a positive control would ideally consist of patients with confirmed malabsorption receiving an intervention with established efficacy, such as liquid levothyroxine formulation or modified administration protocols. However, as a signal-detection investigation, we prioritized comparing vitamin C supplementation against placebo to establish baseline feasibility and preliminary effect size estimation for future definitive trials. The absence of a positive control is acknowledged as a limitation that should be addressed in subsequent larger studies, where the comparison with established interventions may provide valuable benchmarking for assessing the magnitude of vitamin C effects.

#### 2.3.5 Compliance assessment

Compliance was assessed through patient self-report and pharmacy dispensing records verification. While pharmacy records confirmed regular medication dispensing, we did not perform levothyroxine absorption testing as recommended by current European Thyroid Association (ETA) guidelines for patients requiring high doses (29).

### 2.4 Study outcomes

The primary outcomes, assessed at baseline and 16 weeks, were: 1. Change in Zulewski clinical score (a validated 12-item scale, score range 0-12) (30, 31). 2. Change in serum TSH levels. Secondary outcomes included changes in free T4 levels, weight, BMI, and daily levothyroxine dose.

We selected TSH rather than direct thyroid hormone measurement as the primary biochemical endpoint for several evidence-based reasons. First, TSH represents the most sensitive biomarker for assessing thyroid hormone adequacy in primary hypothyroidism, as even subtle changes in circulating thyroid hormone levels produce logarithmic changes in TSH through the hypothalamic-pituitary-thyroid feedback axis. Second, current clinical guidelines from the American Thyroid Association and European Thyroid Association establish TSH normalization as the primary therapeutic target in hypothyroidism management. Third, in patients already receiving levothyroxine therapy, TSH provides the most reliable indicator of whether improved absorption has occurred. Free T4 was measured as a secondary outcome to provide complementary information on peripheral thyroid hormone status.

### 2.5 Laboratory methods

Serum vitamin C levels were measured using a Human Vitamin C ELISA Kit (EK 710149, AFG Bioscience). TSH, T3, and T4 were measured by a chemiluminescent assay (Cobas e411, Roche Diagnostics). Vitamin C insufficiency was defined as <40 µmol/L.

### 2.6 Statistical analysis

Given the pilot nature and small sample size, non-parametric tests were used for primary comparisons. The Wilcoxon signed-rank test was used for within-group comparisons, and the Mann-Whitney U test was used for between-group comparisons. An Analysis of Covariance (ANCOVA) was used to assess the treatment difference on primary outcomes while adjusting for baseline values. An intention-to-treat analysis was not performed as the single participant lost to follow-up had no post-baseline data; therefore, the primary analysis is per-protocol. We calculated 95% confidence intervals for the differences in means and the effect size (Cohen’s d) for primary outcomes. A *post-hoc* power calculation confirmed that with n=11, the study had <20% power to detect a medium effect size, underscoring its signal-detection purpose. A p-value of <0.05 was considered statistically significant. All analyses were performed using SPSS version 30.0.0 (IBM Corp., Armonk, NY).

## 3 Results

### 3.1 Phase 1: vitamin C status assessment

The hypothyroid group (n=26, 26.9% male, mean age 45.3 ± 12.1 years) had lower mean vitamin C levels (59.33 ± 24.62 µmol/L) compared to controls (n=91, 29.7% male, mean age 22.4 ± 3.2 years, vitamin C 73.12 ± 14.03 µmol/L), though this difference was not statistically significant (p=0.21).

Vitamin C insufficiency (<40 µmol/L) was more frequent in hypothyroid patients (19.2%, 5/26) than controls (7.7%, 7/91). Specifically, 15.4% (4/26) had levels between 24-39 µmol/L and 3.8% (1/26) had deficiency (<24 µmol/L) in the hypothyroid group. See **Table 1** for more details.

**Table 1:** Baseline characteristics of participants in the vitamin C (treatment) and oral rehydration salt (placebo) groups (n = 11).

No	Characteristics	Treatment (n=6)	Placebo (n=5)	Total (n=11)
1	Age (in years)	37.67 ± 10.76	49.40 ± 6.30	43.00 ± 10.56
2	Sex	5 females	3 females	8 females
3	Duration of hypothyroidism (years)	12.17 ± 6.52	15.60 ± 3.29	13.73 ± 5.36
4	Weight (kg)	72.67 ± 19.23	100.60 ± 37.98	85.36 ± 31.22
5	Body mass index (kg/m²)	26.55 ± 6.98	43.35 ± 23.92	34.20 ± 18.17
6	Daily levothyroxine dose (µg)	155.36 ± 40.54	190.71 ± 64.96	171.43 ± 53.36
7	Serum thyroid-stimulating hormone level (mU/L)	6.44 ± 3.77	3.79 ± 1.81	5.23 ± 3.22
8	Serum free thyroxine levels (pmol/L)	16.68 ± 1.70	18.28 ± 1.69	17.41 ± 1.81
9	Zulewski clinical score (range from 0 to 12)	5.67 ± 1.51	4.20 ± 1.09	5.00 ± 1.48

Baseline demographic and clinical characteristics of the 11 participants who completed the trial. Data are presented as mean ± standard deviation or as frequencies. Despite randomization, the small sample size resulted in significant baseline imbalances, most notably in age and Body Mass Index (BMI). This imbalance represents a critical limitation of the study, as it compares two metabolically distinct populations (overweight vs. morbidly obese).

### 3.2 Phase 2: pilot RCT results

#### 3.2.1 Participant flow and baseline characteristics

**Figure 1:**
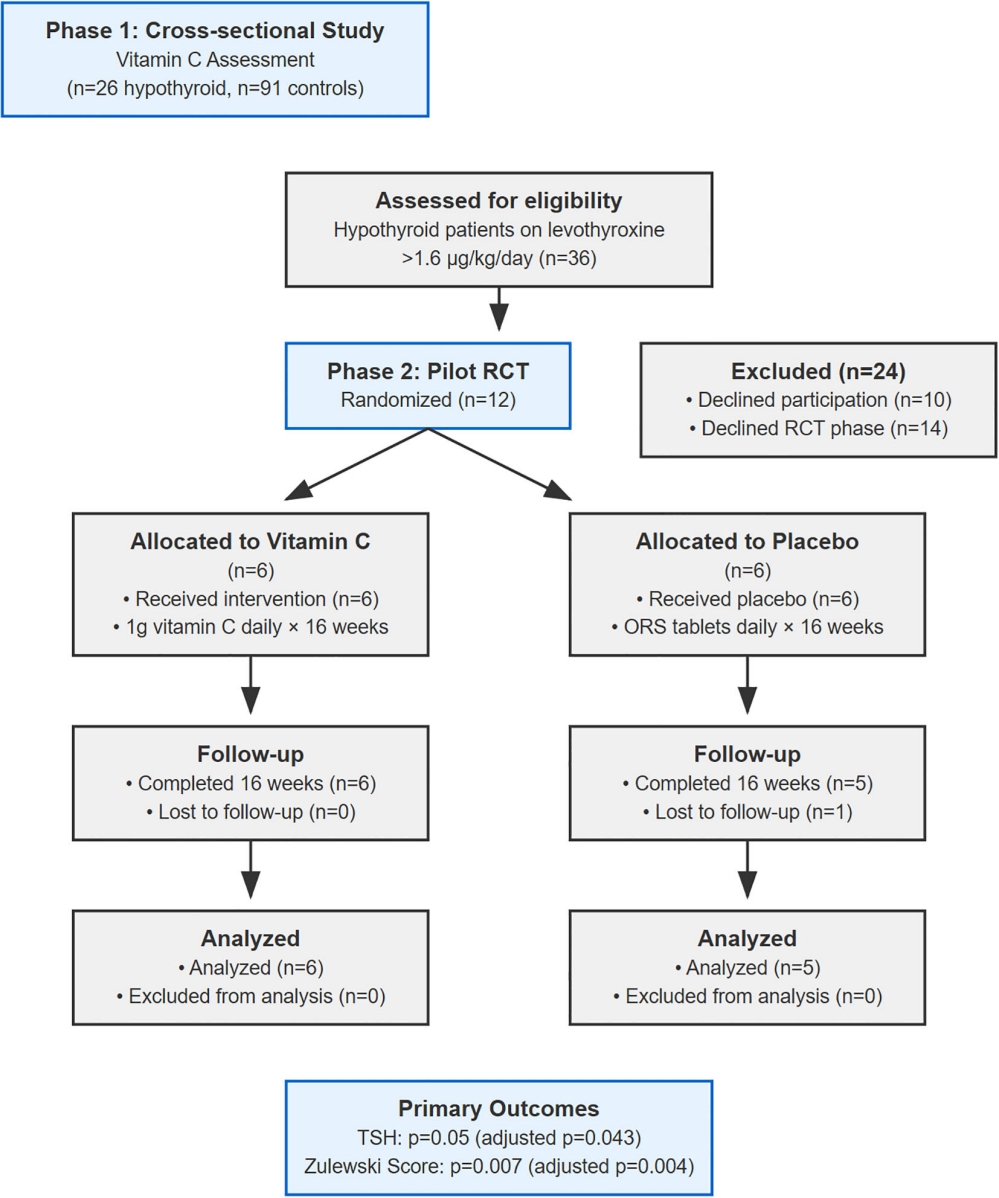
CONSORT flow diagram for pilot proof-of-concept pilot study.

Twelve patients were randomized (6 per group). One placebo group participant was lost to follow-up, leaving 11 for analysis. Baseline characteristics showed significant imbalances despite randomization (**Table 1**), a known risk in small trials. See **Figure 1** for patient flow details.

Notably, the placebo group had a substantially higher BMI (mean 43.4 vs 26.6 kg/m²), corresponding to morbid obesity versus overweight. The placebo group’s BMI also showed extreme variability (SD = 23.92), with one patient (BMI 70.3 kg/m²) accounting for most of the between-group difference. This confirmed that this was not a systematic imbalance but rather the unfortunate random allocation of one extreme case to a small group (n=5).The mean weight-adjusted levothyroxine dose was 2.14 µg/kg/day in the treatment group and 1.90 µg/kg/day in the placebo group. Individual patient data are presented in **Supplementary Table S1** to provide complete transparency regarding the distribution of baseline characteristics and treatment responses. **Supplementary Figure S2** Panel C illustrates the relationship between baseline BMI and treatment response and indicates how one outlier patient substantially influenced group-level statistics.

#### 3.2.2 Primary outcomes

After 16 weeks, the vitamin C group showed a greater reduction in the Zulewski Clinical Score compared to the placebo group (mean reduction of 5.00 ± 0.89 vs 1.40 ± 1.34 points; mean difference 3.60, 95% CI: 1.88 to 5.32; p=0.007). A greater reduction was also observed in TSH levels (mean reduction of 4.08 ± 3.16 vs 2.35 ± 1.24 mU/L; mean difference 1.73, 95% CI: -2.14 to 5.60; p=0.05). After adjusting for baseline values via ANCOVA, the difference between groups remained statistically significant for the Zulewski score (adjusted p=0.004) but only marginally significant for TSH (adjusted p=0.043). See **Table 2** for details.

**Table 2:** Changes in clinical/biochemical variables from pre-intervention to post-16-week intervention in the vitamin C (treatment) and placebo groups.

Sr. No	Changes in variables pre- and post-16-week intervention	Treatment (n=6)	Placebo (n=5)	Test statistic (Z-score)	p-value (Mann-Whitney U)
1	Weight (kg)	-0.34 ± 0.49	12.8 ± 0.50	-1.051	0.29
2	Body mass index (kg/m²)	-0.15 ± 0.08	6.66 ± 7.32	-0.980	0.33
3	Daily levothyroxine dose (µg)	-6.55 ± 6.44	7.86 ± 6.25	-0.365	0.72
4	Serum thyroid-stimulating hormone level (mU/L)	-4.08 ± 3.16	-2.35 ± 1.24	-1.956	0.05*
5	Serum free thyroxine levels (pmol/L)	2.20 ± 5.49	0.74 ± 3.27	-1.335	0.18
6	Zulewski clinical score (range from 0 to 12)	-5.00 ± 0.89	-1.40 ± 1.34	-2.675	0.007*

Changes in clinical and biochemical variables from baseline to 16 weeks. Values represent mean change ± standard deviation. The p-values shown are from the unadjusted Mann-Whitney U test for the comparison of change between the treatment and placebo groups. p<0.05. After adjusting for baseline values using ANCOVA, the between-group difference remained statistically significant for the Zulewski score (adjusted p=0.004) only marginally significant for TSH (adjusted p=0.043).

#### 3.2.3 Sensitivity analysis

To explore the impact of the profound BMI imbalance, we conducted a *post-hoc* sensitivity analysis, excluding the one outlier participant with a BMI of 70.3 kg/m² from the placebo group. The BMI difference when outlier was excluded turned out to be 4.87 (26.55 vs 31.42) kg/m². In this analysis (n=10), the between-group difference in Zulewski score reduction remained statistically significant (p=0.02), while the difference in TSH change was attenuated (p=0.09). This analysis suggests the observed difference in clinical symptoms was robust, but it also highlights the confounding influence of obesity. See **Supplementary Material** for more details.

#### 3.2.4 Safety

No adverse events were reported in either group.

## 4 Discussion

This proof-of-concept pilot randomized controlled trial successfully demonstrates the feasibility of investigating vitamin C supplementation as an adjunct therapy in hypothyroid patients requiring high-dose levothyroxine. More importantly, it identifies a potential therapeutic signal that justifies further investigation through adequately powered trials. To our knowledge, this represents the first controlled trial investigating this intervention in any population and the first vitamin C assessment in Middle Eastern hypothyroid patients.

The paradoxically high weight-adjusted levothyroxine doses (1.9-2.1 µg/kg/day versus an expected ~1.1 µg/kg/day in obesity), confirmed through pharmacy dispensing records, strongly suggest our population had unidentified factors affecting absorption despite our exclusion criteria. As noted in the 2025 ETA Guidelines, such patients represent a challenging subgroup where alternative strategies like vitamin C supplementation merit investigation (29). The fact that these high doses were consistently dispensed and taken, yet patients remained suboptimally controlled, supports genuine malabsorption rather than non-compliance as the primary issue.

### 4.1 Proof-of-concept achieved

This study successfully achieves its primary objective as a proof-of-concept investigation. The observed improvements in both Zulewski clinical score and TSH levels provide the “signal” that justifies proceeding to definitive trials. Importantly, we demonstrated feasibility in recruitment, retention (91.7%), intervention delivery, and outcome assessment, all critical elements for proof-of-concept studies.

The effect sizes observed (TSH reduction difference of 1.73 mU/L between groups) provide crucial data for powering future definitive trials. Using these pilot data, a definitive trial would require approximately 35–40 patients per group to achieve 80% power, a feasible sample size for multicenter collaboration. This sample size calculation represents a key deliverable of proof-of-concept research.

### 4.2 Mechanistic insights from proof-of-concept findings

Our study was not designed to elaborate on mechanistic insights, and we emphasize that any mechanistic discussion remains entirely speculative without the supporting biomarker data. While vitamin C theoretically can enhance levothyroxine absorption by lowering gastric pH, our deliberate use of a pH-matched placebo (pH difference only 0.15 units) suggests that if the observed signal represents a genuine effect, non-pH mechanisms may contribute. Alternative hypotheses include potential antioxidant effects that may be beneficial in autoimmune thyroiditis, a condition characterized by elevated oxidative stress, or effects on intestinal transport mechanisms. However, without direct measurement of oxidative stress markers, levothyroxine pharmacokinetics, or intestinal absorption parameters, we cannot distinguish between these proposed mechanisms. This mechanistic uncertainty does not diminish the value of this proof-of-concept work but clearly defines critical measurements that must be incorporated into future definitive trials.

### 4.3 Vitamin C insufficiency in hypothyroidism

The higher prevalence of vitamin C insufficiency in hypothyroid patients (19.2% vs 7.7%) represents a novel finding requiring confirmation. This could reflect dietary differences, increased oxidative stress in hypothyroidism, or altered vitamin C metabolism. The 19.2% insufficiency rate contrasts sharply with the 1.4% reported in other high-income countries (32), suggesting population-specific factors. The patients in our study were all Emiratis, and although iodine levels were not assessed, the UAE population generally maintains adequate iodine status according to regional studies (33). This disparity in vitamin C insufficiency found in Emirati patients with hypothyroidism when compared with other population studies may suggests local population-specific factors which may include regional dietary patterns characterized by variable fresh fruit and vegetable consumption, traditional food preparation methods that could reduce vitamin C content through heating or prolonged storage, and environmental factors such as the region’s hot climate which may affect both dietary preferences and nutrient stability in foods. Additionally, cultural meal timing patterns and the interaction between fasting periods and vitamin status merit consideration. However, we must emphasize that without detailed dietary assessment, food frequency questionnaires, or assessment of traditional versus modern dietary patterns in our study population, these explanations remain hypothetical. Future research should include comprehensive nutritional assessment to understand whether vitamin C insufficiency represents a modifiable risk factor specific to this population.

### 4.4 Strengths and limitations

This study’s strengths lie in its rigorous proof-of-concept design, including randomization, double-blinding, and the use of a pH-matched placebo. However, the limitations are significant and must be considered when interpreting the results. We have structured them hierarchically from most to least critical:

#### 4.4.1 Baseline imbalances

The most significant limitation was the failure of randomization to achieve baseline balance, resulting in profound differences in age and, most critically, BMI. This compares two metabolically distinct groups (overweight vs. morbidly obese) and severely confounds the interpretation of the results. The imbalance was driven primarily by one outlier patient (BMI 70.3 kg/m²) in the placebo group, demonstrating the vulnerability of small trials to extreme values.

#### 4.4.2 Sample size and power

The very small sample size (n=11) renders the study severely underpowered (<20% power for medium effects) and precludes any definitive conclusions about efficacy. The findings should be viewed as hypothesis-generating only.

#### 4.4.3 Screening limitations

The absence of systematic screening for occult malabsorption conditions (e.g., subclinical celiac disease, H. pylori) is a major limitation that may explain the high levothyroxine requirements observed.

#### 4.4.4 Absence of positive control

The study design did not include a positive control group receiving an established intervention for levothyroxine malabsorption, such as liquid levothyroxine formulation or modified administration protocols. While appropriate for initial proof-of-concept research, this limits our ability to benchmark the magnitude of any vitamin C effect against interventions with known efficacy. Future trials should consider three-arm designs comparing vitamin C supplementation, established interventions, and placebo to provide this important context.

#### 4.4.5 Age-mismatched controls

In Phase 1, the 23-year age difference between groups invalidates direct comparison of vitamin C status and serves only as internal hypothesis-generation.

#### 4.4.6 Lack of mechanistic markers

The study did not include measures of levothyroxine absorption or oxidative stress, making any discussion of mechanism speculative.

#### 4.4.7 Limited generalizability

This study was conducted exclusively in Emirati patients with autoimmune hypothyroidism in a single-center setting within the United Arab Emirates healthcare system. These findings cannot be extrapolated to other populations, particularly those with different ethnic backgrounds, dietary patterns, genetic polymorphisms affecting thyroid hormone metabolism or vitamin C transport, or alternative causes of hypothyroidism such as post-ablative or post-surgical states. The homogeneous population, while reducing some sources of variability, simultaneously restricts the applicability of findings. The absence of racial, ethnic, and geographic diversity represents a significant limitation that must be addressed through future multi-centered international trials. Additionally, the specific characteristics of our population requiring high-dose levothyroxine may not represent typical hypothyroid patients, further limiting generalizability to broader clinical populations.

### 4.5 Clinical context and future directions

Our study addresses a clinically challenging subgroup of patients requiring high-dose levothyroxine, a population highlighted as needing alternative management strategies in recent ETA guidelines (29). The primary value of this proof-of-concept research is not to guide current practice but to rigorously inform the design of a definitive trial. Based on the limitations identified in this pilot work, we can now specify essential design elements for a definitive trial. These include: stratified randomization by BMI category and baseline TSH level to prevent the imbalances observed here; systematic screening for occult malabsorption including celiac serology, Helicobacter pylori testing, and consideration of levothyroxine absorption testing as recommended by 2025 ETA Guidelines; inclusion of a positive control arm receiving liquid levothyroxine or other established intervention; measurement of mechanistic biomarkers including oxidative stress markers, inflammatory cytokines, and pharmacokinetic assessment of levothyroxine absorption; comprehensive dietary assessment including vitamin C intake and overall nutritional status; longer follow-up duration of at least 6 to 12 months to assess durability of any effects; and multicenter international design to ensure diverse population representation. The sample size calculations derived from our pilot data suggest approximately 35 to 40 patients per group would provide 80% power to detect clinically meaningful differences, making such a trial feasible within reasonable resource constraints.

## 5 Conclusions

This pilot RCT suggests that vitamin C supplementation warrants investigation for potential effects on clinical and biochemical outcomes in hypothyroid patients requiring high-dose levothyroxine. However, the findings are highly preliminary and must be interpreted with extreme caution due to critical limitations, including a very small sample size and severe baseline imbalances. The study successfully demonstrates the feasibility of conducting such a trial and provides essential data including effect size estimates and identification of critical design requirements to inform a future, adequately powered, multicenter trial. Such a definitive trial must incorporate stratified randomization by BMI and baseline TSH, include positive controls with established interventions, measure mechanistic biomarkers, assess dietary and nutritional status comprehensively, and ensure diverse population representation. Until such confirmation is obtained, these preliminary findings should be considered hypothesis-generating only and should not influence clinical decision-making or treatment recommendations.

## Data Availability

The original contributions presented in the study are included in the article/**Supplementary Material**. Further inquiries can be directed to the corresponding authors.
