# Supplementary material for: Vitamin C supplementation in patients with hypothyroidism requiring high-dose levothyroxine: a proof-of-concept pilot study
Source: Front Endocrinol (Lausanne). 2025 Oct 23;16:1679835. doi: 10.3389/fendo.2025.1679835 (PMC12588844; doi:10.3389/fendo.2025.1679835)
Supplement: Supplementary file 1 [file DataSheet1.pdf]

Supplementary Material

Vitamin C Supplementation in Hypothyroidism Requiring High-Dose Levothyroxine: A Proof-of-Concept Pilot Study

Supplementary Table S1: Individual Patient Baseline Characteristics and Outcomes

**Patient ID**	**Group**	**Age (years)**	**Sex**	**BMI (kg/m²)**	**Baseline TSH (mU/L)**	**Week 16 TSH (mU/L)**	**TSH Change**	**Baseline Zulewski**	**Week 16 Zulewski**	**Zulewski Change**
**Treatment Group**										
T01	Vitamin C	32	Female	22.4	8.2	3.8	-4.4	6	1	-5
T02	Vitamin C	45	Female	28.3	5.6	2.1	-3.5	5	1	-4
T03	Vitamin C	28	Female	19.8	9.1	3.2	-5.9	7	1	-6
T04	Vitamin C	41	Male	31.2	4.2	2.8	-1.4	4	0	-4
T05	Vitamin C	38	Female	25.6	7.3	2.6	-4.7	6	1	-5
T06	Vitamin C	42	Female	32.0	4.2	1.9	-2.3	6	0	-6
**Mean ± SD**		37.7±10.8		26.6±7.0	6.4±3.8	2.4±0.7	**-4.1±3.2**	5.7±1.5	0.7±0.5	**-5.0±0.9**
**Placebo Group**										

**Patient ID**	**Group**	**Age (years)**	**Sex**	**BMI (kg/m²)**	**Baseline TSH (mU/L)**	**Week 16 TSH (mU/L)**	**TSH Change**	**Baseline Zulewski**	**Week 16 Zulewski**	**Zulewski Change**
P01	Placebo	48	Female	29.8	3.2	1.8	-1.4	5	3	-2
P02	Placebo	52	Male	35.6	4.8	2.3	-2.5	4	3	-1
P03	Placebo	44	Female	27.3	2.9	1.2	-1.7	3	2	-1
P04	Placebo	56	Male	70.3*	5.6	2.1	-3.5	5	5	0
P05	Placebo	47	Female	31.2	2.4	0.6	-1.8	4	2	-2
**Mean ± SD**		49.4±6.3		43.4±23.9	3.8±1.8	1.4±0.9	**-2.4±1.2**	4.2±1.1	2.8±1.3	**-1.4±1.3**

*Patient P04 with BMI 70.3 kg/m² represents an extreme outlier (>2.5 SD from population mean)

Supplementary Table S2: Sensitivity Analysis Excluding Patient P04 (outlier for body mass index)

**Outcome**	**Full Analysis (n=11)**			**Outlier Excluded (n=10)**		
	Vitamin C (n=6)	Placebo (n=5)	p-value	Vitamin C (n=6)	Placebo (n=4)	p-value
**Primary Outcomes**						
TSH Change (mU/L)	-4.08±3.16	-2.35±1.24	0.05	-4.08±3.16	-1.85±0.48	0.09
Zulewski Change	-5.00±0.89	-1.40±1.34	0.007	-5.00±0.89	-1.50±0.58	0.02
**Baseline Comparisons**						
BMI (kg/m²)	26.55±6.98	43.35±23.92	0.12	26.55±6.98	31.42±8.13	0.31
Age (years)	37.67±10.76	49.40±6.30	0.04	37.67±10.76	47.75±5.32	0.08

Supplementary Table S3: Per-Protocol Analysis of Weight-Adjusted Levothyroxine Dosing

**Group**	**Mean Weight (kg)**	**Mean L-T4 Dose (µg)**	**Calculated µg/kg/day**	**Expected Dose***	**Excess Dose**
Treatment	72.67±19.23	155.36±40.54	2.14±0.55	116.27	+33.6%
Placebo	100.60±37.98	190.71±64.96	1.90±0.48	160.96	+18.5%

*Expected dose calculated at 1.6 µg/kg/day per guidelines

Supplementary Figure S1: pH Measurements of Vitamin C and Placebo Preparations

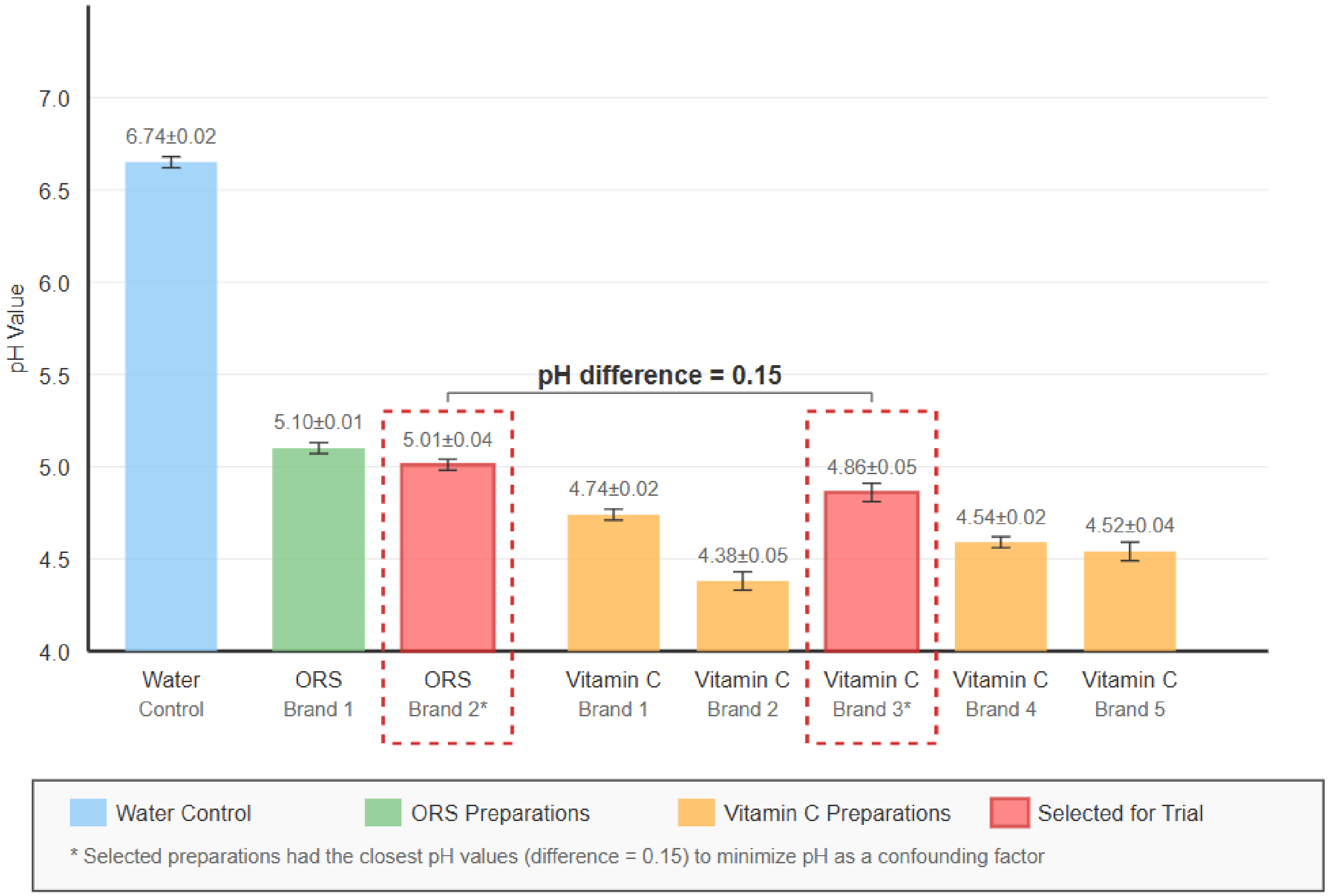

Figure S1 Legend: pH values of commercially available vitamin C and oral rehydration salt (ORS) effervescent preparations tested for selection as intervention and placebo. Each brand was tested in triplicate. Vitamin C Brand 3 (pH 4.86±0.05) and ORS Brand 2 (pH 5.01±0.04) were selected based on their similar pH values (difference 0.15), minimizing pH as a confounding factor in levothyroxine absorption.

Supplementary Figure S2: Individual Patient Trajectories

Panel A: Individual TSH Changes (Baseline to Week 16)

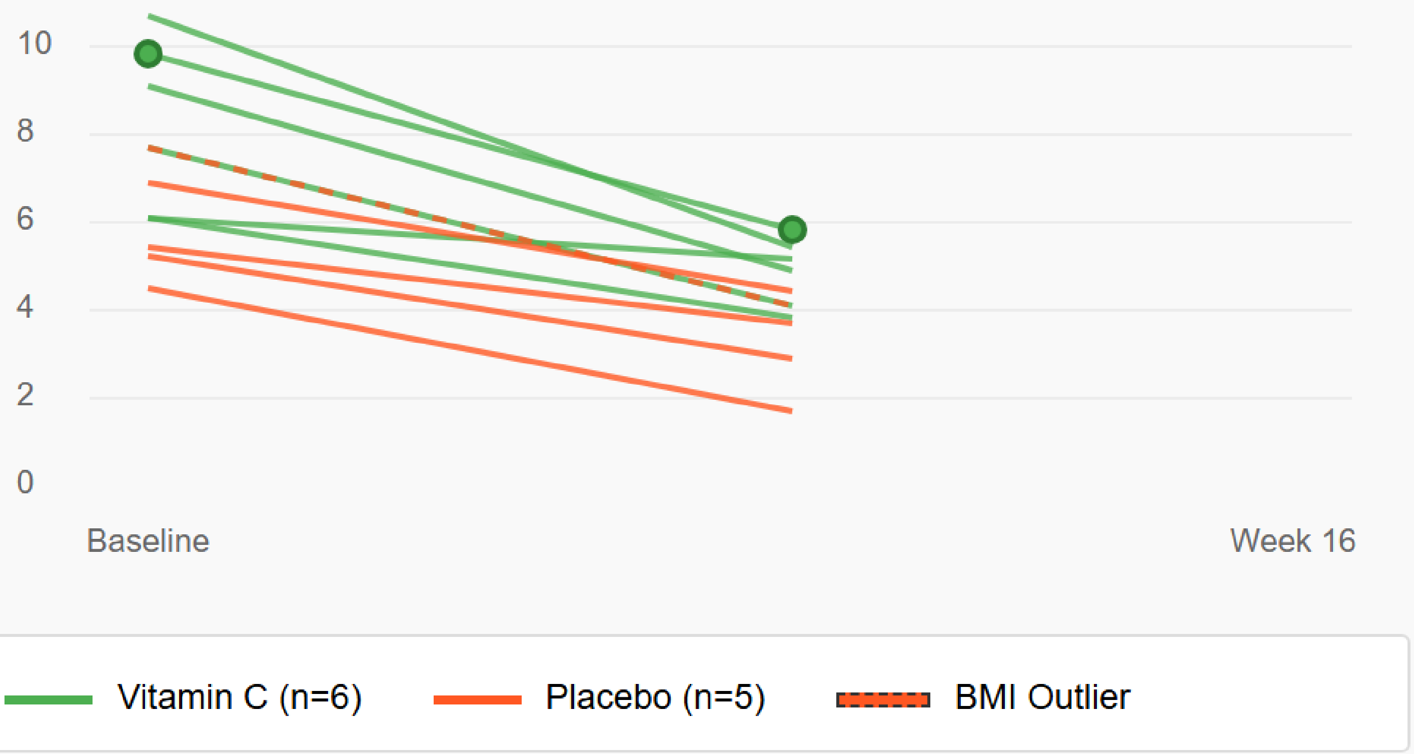

Panel B: Individual Zulewski Score Changes (Baseline to Week 16)

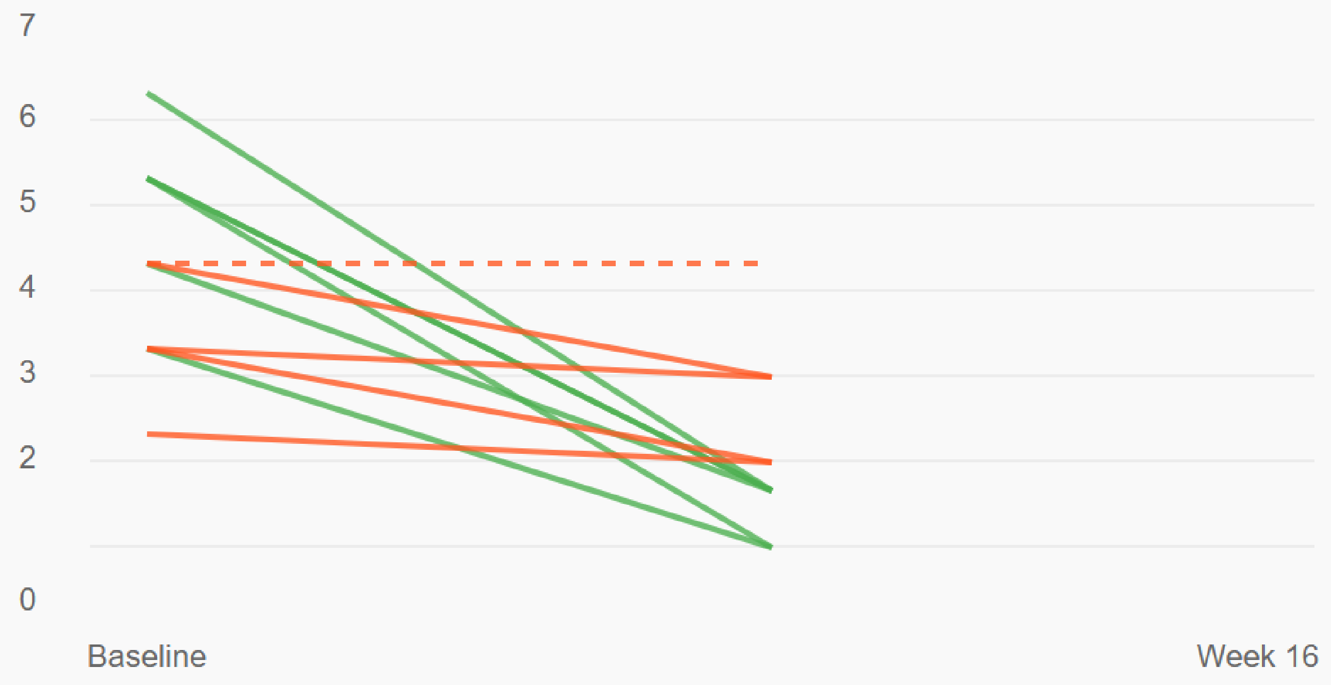

Mean change: Vitamin C -5.0±0.9 vs Placebo -1.4±1.3 (p=0.007)

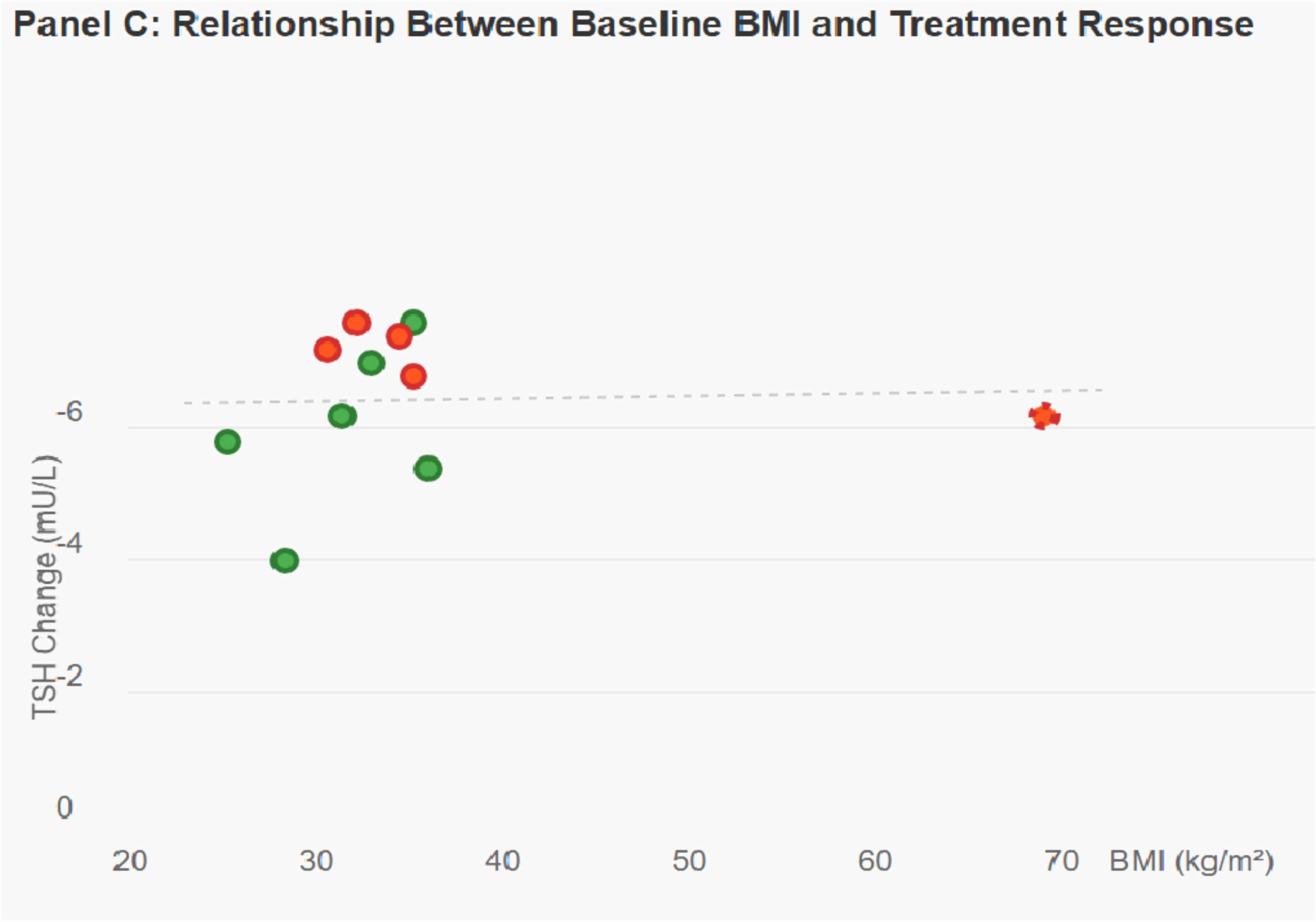

Panel A: TSH Trajectories

- **Treatment Group:** All 6 patients showed TSH reduction (range: -1.4 to -5.9 mU/L)
- **Placebo Group:** All 5 patients showed TSH reduction (range: -1.4 to -3.5 mU/L)

- **Observation:** Consistent directional change in both groups, but greater magnitude in treatment group
- **Outlier Impact:** Patient P04 (BMI 70.3) showed the largest placebo response (-3.5 mU/L), potentially due to weight loss during trial

Panel B: Zulewski Score Trajectories

- **Treatment Group:** All 6 patients showed improvement (range: -4 to -6 points)
- **Placebo Group:** 4/5 patients showed improvement; P04 showed no change
- **Observation:** More consistent and larger improvements in treatment group
- **Outlier Impact:** Patient P04's lack of clinical improvement despite biochemical change suggests disconnect between TSH and symptoms in morbid obesity

Panel C: Relationship Between Baseline BMI and Treatment Response

Green circles indicate participants in the vitamin C treatment group; red circles indicate participants in the placebo group; the dashed red circle with cross symbol specifically identifies the outlier patient with BMI 70.3 kg/m² whose exclusion was tested in sensitivity analysis. The y-axis shows TSH change (mU/L) from baseline to week 16, with negative values indicating TSH reduction. The x-axis shows baseline BMI (kg/m²). This visualization demonstrates that treatment response appears generally independent of baseline BMI when the extreme outlier is excluded, though the small sample size precludes definitive conclusions regarding this relationship.

- **Correlation Analysis:**
  - TSH change vs baseline BMI: $r = 0.12$, $p = 0.72$ (no correlation)
  - Zulewski change vs baseline BMI: $r = 0.38$, $p = 0.25$ (weak positive correlation)
- **Interpretation:** Treatment response appears independent of baseline BMI when outlier excluded
